# Variable viral clearance despite adequate ganciclovir plasma levels during valganciclovir treatment for cytomegalovirus disease in D+/R- transplant recipients

**DOI:** 10.1186/1471-2334-10-2

**Published:** 2010-01-06

**Authors:** Nancy Perrottet, Oriol Manuel, Frédéric Lamoth, Jean-Pierre Venetz, Roland Sahli, Laurent A Decosterd, Thierry Buclin, Manuel Pascual, Pascal Meylan

**Affiliations:** 1 Division of Clinical Pharmacology and Toxicology, University Hospital (CHUV), Lausanne, Switzerland; 2 Organ Transplantation Centre, University Hospital (CHUV), Lausanne, Switzerland; 3 Microbiology Institute, University Hospital (CHUV), Lausanne, Switzerland

## Abstract

**Background:**

Valganciclovir, the oral prodrug of ganciclovir, has been demonstrated equivalent to iv ganciclovir for CMV disease treatment in solid organ transplant recipients. Variability in ganciclovir exposure achieved with valganciclovir could be implicated as a contributing factor for explaining variations in the therapeutic response. This prospective observational study aimed to correlate clinical and cytomegalovirus (CMV) viral load response (DNAemia) with ganciclovir plasma concentrations in patients treated with valganciclovir for CMV infection/disease.

**Methods:**

Seven CMV D+/R- transplant recipients (4 kidney, 2 liver and 1 heart) were treated with valganciclovir (initial dose was 900-1800 mg/day for 3-6.5 weeks, followed by 450-900 mg/day for 2-9 weeks). DNAemia was monitored by real time quantitative PCR and ganciclovir plasma concentration was measured at trough (C_trough_) and 3 h after drug administration (C_3h_) by HPLC.

**Results:**

Four patients presented with CMV syndrome, two had CMV tissue-invasive disease after prophylaxis discontinuation, and one liver recipient was treated pre-emptively for asymptomatic rising CMV viral load 5 weeks post-transplantation in the absence of prophylaxis. CMV DNAemia decreased during the first week of treatment in all recipients except in one patient (median decrease: -1.2 log copies/mL, range: -1.8 to 0) despite satisfactory ganciclovir exposure (AUC_0-12 _= 48 mg·h/L, range for the 7 patients: 40-118 mg·h/L). Viral clearance was obtained in five patients after a median of time of 34 days (range: 28-82 days). Two patients had recurrent CMV disease despite adequate ganciclovir exposure (65 mg·h/L, range: 44-118 mg·h/L).

**Conclusions:**

Valganciclovir treatment for CMV infection/disease in D+/R- transplant recipients can thus result in variable viral clearance despite adequate ganciclovir plasma concentrations, probably correlating inversely with anti-CMV immune responses after primary infection.

## Background

Cytomegalovirus (CMV) used to rank first as a cause for morbidity and mortality among solid organ transplants (SOT) recipients [1]. CMV disease can be prevented either by CMV prophylaxis or pre-emptive treatment guided by CMV viral load monitoring [1]. CMV-seronegative recipients who receive a transplant from a CMV-positive donor (D+/R-) are at highest risk of developing late CMV disease despite prophylaxis [2] with an incidence up to 43% [3]. Valganciclovir, the ester prodrug of ganciclovir, is currently used for CMV prophylaxis [4] and has been demonstrated equivalent to iv ganciclovir for CMV disease treatment in SOT recipients [5]. As ganciclovir plasma levels were not reported in most treatment studies using valganciclovir, variability in ganciclovir exposure achieved with this oral prodrug (e.g. due to malabsorption) could be imagined as a contributing factor partly explaining variations in the therapeutic response. The present prospective study aimed at describing the clinical and virological outcome (CMV viral load response) along with ganciclovir plasma concentration exposure in SOT patients receiving valganciclovir treatment for CMV infection/disease.

## Methods

### Patients

The present consecutive series of patients presenting CMV disease and treated with valganciclovir was observed during a population pharmacokinetic study of valganciclovir conducted at the University Hospital, Lausanne, Switzerland, with the approval of the local ethics committee [6]. Adult SOT recipients with either CMV asymptomatic infection treated pre-emptively or with CMV disease [7] receiving oral valganciclovir treatment were enrolled after giving a written informed consent. Valganciclovir therapeutic dosage for CMV infection was 900 mg twice daily (adjusted to renal function according to the manufacturer recommendations and subsequently to ganciclovir blood levels) followed by a maintenance therapy (900 mg once daily with similar adjustment). Ganciclovir levels were measured weekly at trough (C_trough_) and 3 hours after oral administration (C_3 h_) during treatment along with CMV viral load. The duration of the therapy was left to the decision of the physician in charge of the patient. In case of recurrence of CMV disease (defined as second episode after first CMV disease symptoms resolution), valganciclovir was reintroduced at therapeutic dosage. Kidney and cardiac transplant recipients received induction therapy with basiliximab (n = 3) or thymoglobuline (n = 2). The maintenance immunosuppressive regimen included prednisone in all patients associated with either tacrolimus (in 5 patients) or cyclosporine (in 2 patients), and mycophenolate mofetil (in 4 patients), mycophenolate sodium (in 1 patient) or azathioprine (in 1 patient).

### Ganciclovir plasma level and pharmacokinetic profile

Plasma ganciclovir concentrations were determined by reverse-phase HPLC coupled with spectrofluorimetric detection according to a validated method [8]. The calibration curve was linear between 0.1 and 10 mg/L, the inter-day coefficient of variation was lower than 3.5% and the range of inter-day deviations comprised within -0.4 to +1.4%.

Ganciclovir plasma concentration results were analysed in a population pharmacokinetic study in the whole population of 65 transplant patients including this subgroup along with a majority of patients receiving valganciclovir for CMV prophylaxis. The analysis was performed by non-linear mixed effect modelling using the NONMEM^® ^computer program. The structural model was two-compartment with first-order absorption. Systemic clearance was markedly influenced by GFR, sex and graft types. Body weight and sex influenced central volume of distribution. There was no difference in drug disposition between patients receiving valganciclovir for prophylaxis versus therapy. Ganciclovir exposure was evaluated by calculating the area under the curve (AUC) for each individual and sampling occasion, based on the subject-specific clearance value estimated at the end of the population analysis (maximum likelihood a posteriori Bayesian estimation) [6].

### Virological and immunity monitoring

CMV viremia was measured in whole blood using a CMV DNA real time quantitative PCR [9] with results expressed in number of copies/mL (limit of quantification: 1000 copies/mL, threshold of detection close to 100 copies/mL). DNAemia clearance was defined after one negative PCR. Recurrence of CMV disease was defined as reappearance in the blood of CMV DNA accompanied by symptoms.

CMV and EBV antibody status of donor and recipient were determined using commercialised kit. CMV specific and EBV specific T-cell response were assessed by ELISPOT assays. (Additional file 1)

Mutations in the CMV UL97 kinase gene were looked for in one patient. Part of the UL97 region covering most of the known mutations associated with resistance to ganciclovir (codons 437-609 [10,11]) was amplified by PCR using CMV_UL97 M_F (TGCACGTTGGCCGACGCTAT: position 1308-1327 within the UL97 open reading frame) and CMV_UL97 M_R (GCCGCCAGAATGAGCAGACA position 1837-1818 on the complementary strand of the UL97 open reading frame). (Additional file 1)

## Results

**Figure 1:**
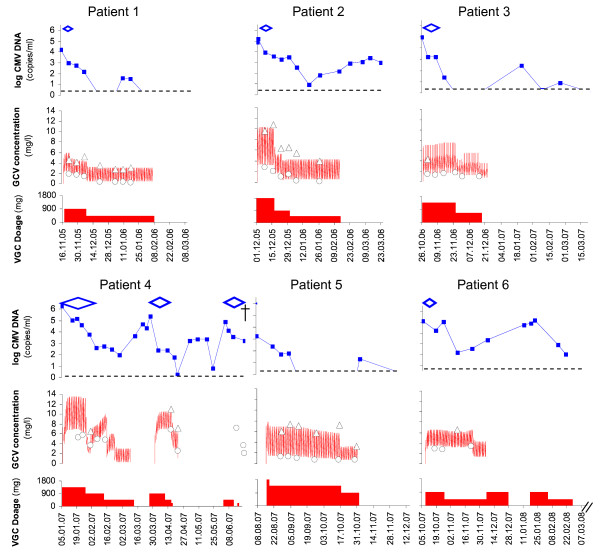
**CMV treatment with valganciclovir in patients 1-6**. valganciclovir dosage (red rectangle), ganciclovir plasma concentration (closed white circle: concentration measured at trough, closed white triangle: concentration measured 3 h after last dose, red line: concentration predicted by population pharmacokinetic model), CMV viremia (blue square and solid blue line) and symptoms period (blue diamond)

**Figure 2:**
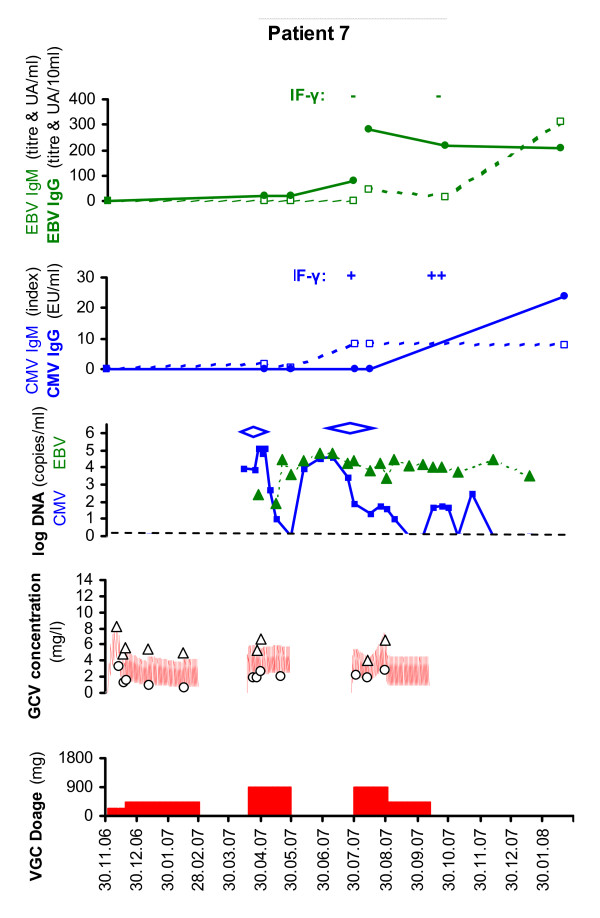
**CMV prophylaxis and treatment with valganciclovir in patient 7**. valganciclovir dosage (red rectangle), ganciclovir plasma concentration (closed white circle: concentration measured at trough, closed white triangle: concentration measured 3 h after last dose, red line: concentration predicted by population pharmacokinetic model), CMV viremia (blue square and solid blue line), symptoms period (blue diamond), EBV viremia (green triangle and dotted green line), anti-CMV IgM and IgG (IgM: closed white square and dotted blue line, IgG: blue circle and solid blue line), anti-EBV IgM and IgG (IgM: closed white square and dotted green line, IgG: closed green circle and solid green line), CMV specific T-cell response and EBV specific T-cell response (Interferon-γ, IF-γ -: negative; IF-γ +: positive)

Seven CMV D+/R- patients (4 kidney, 2 liver and 1 heart recipients) were treated with valganciclovir: 6 for late-onset CMV disease (CMV syndrome n = 4 and CMV tissue-invasive disease n = 2) and one pre-emptively for CMV asymptomatic infection (n = 1) (Table 1). Treatment started between 1.4 and 21.5 months after discontinuation of valganciclovir prophylaxis in 6 patients and 5 weeks after transplantation in the liver recipient who was treated pre-emptively. Initial valganciclovir dose was 900-1800 mg/day adjusted to calculated creatinine clearance for a median of 38 days (range: 20-63 days), followed by maintenance therapy (450-900 mg/day for a median of 27 days, range: 0-62 days). During valganciclovir treatment, maintenance immunosuppressive regimen consisted in prednisone 5-15 mg per day (depending on the time post-transplantation), in tacrolimus (mean plasma trough levels between 6-10 μg/L) in 5 patients and cyclosporine (mean plasma trough levels between 170-250 μg/L) in 2 patients. Doses of mycophenolate mofetil were reduced in 4 patients (initially 1-2 grams per day to 0.5-1 gram per day when valganciclovir was initiated and further stopped in 2 patients), as well as mycophenolate sodium in 1 patient (360 mg per day then stopped) and azathioprine in 1 patient (150 mg per day progressively reduced over 2 months then stopped). Figures 1 and 2 show valganciclovir dosage, ganciclovir plasma levels and CMV viral load over time for each patient.

**Table 1:** Patients characteristics, timing and type of CMV infection/disease and of recurrence

#	Age	Sex	Graft type *date*	Diagnostic	CMV Prophylaxis	Interval^a ^months	CMV disease	Log CMV DNA change/1^st ^week	Clearance of CMV viremia (Time to clearance)	Recurrence of CMV disease *(time from end of therapy)*	Immunosuppressive maintenance regimen^b^
**1**	64	M	Liver	Cirrhosis Child C	VGC^c^	8.9	Colitis	-1.2	Yes *(34 days)*	No	tacrolimus (10 μg/l)^d^
			*19.02.2005*	Alpha-antitrypsin deficiency	450 mg bid		(definite)				prednisone (7.5 qd)
					6 months						MMF^e ^(stop for 1.5 months)
**2**	53	F	Cardiac	Idiopathic dilated	VGC	6.2	Colitis	-1.3	No	No	cyclosporine (200 μg/l)^d^
			*26.05.2005*	cardiomyopathy	450 mg bid		(definite)				prednisone (10 mg qd)
					3 months						MMF^e ^(1000 mg bid)
**3**	46	M	Kidney	Drug toxicity:	VGC	24.5	Syndrome	-1.8	Yes *(29 days)*	No	tacrolimus (7 μg/l)^d^
			*11.10.2004*	cisplatin, ifosfamide, contrast	450 mg qd		(probable)				prednisone (5 mg qd)
				products, non steroidal analgesics	3 months						MPS^f ^(180 mg bid)
**4**	62	M	Kidney	Hepatorenal polykystosis	VGC	9.4	Syndrome	-1.2	No	Syndrome (probable)	tacrolimus (8 μg/l)^d^
			*24.03.2006*		450 mg qd		(probable)			*(21 days)*	prednisone (5 mg qd)
					3 months					Colitis (definite) *(45 days)*	MMF^e ^(500 mg bid)
**5**	49	M	Liver	Cirrhosis Child A	None	1.4	Asymptomatic	-0.8	Yes *(28 days)*	No	cyclosporine (240 μg/l)^d^
			*03.07.2007*	Hepatocellular carcinoma (HCV)			infection				prednisone (15 mg qd)
											AZA^g ^(150 mg qd)
**6**	64	M	Kidney	Hepatorenal polykystosis	VGC	9.7	Syndrome ^h^	-0.8	Yes *(82 days)*	No	tacrolimus (8 μg/l)^d^
			*13.12.2006*		450 mg qd		(probable)				prednisone (10 mg qd)
					3 months						MMF^e ^(750 mg bid)
**7**	68	M	Kidney	Hypertension	VGC	4.4	Syndrome	0	Yes *(42 days)*	Gastritis (definite)	tacrolimus (7 μg/l)^d^
			*01.12.2006*		450 mg qd		(probable)			*(62 days)*	prednisone (5 mg qd)
					3 months						MMF^e ^(250 mg bid)

^a^Interval of time between transplantation and start of valganciclovir treatment

^b^Immunosuppressive maintenance regimen when valganciclovir treatment was introduced

^c^VGC = valganciclovir

^d^trough concentration

^e^MMF = mycophenolate mofetil

^f^MPS = mycophenolic sodium

^g^AZA = azathioprine

^h^This patient received a shorter treatment because of serum creatinine increase (dehydration, diuretic and nephrotoxic drugs). The treatment was reintroduced 10 days after interruption.

Clinical symptoms resolved in all symptomatic patients with a median of 14.5 days of treatment (range: 5-28 days). The median viral load decline in these 7 patients was -1.2 log copies/mL after 1 week of treatment with a wide variation ranging from 0 to -1.8 log copies/mL. Viral clearance was obtained in five patients after a median time of 34 days (range: 28-82 days). In one patient, CMV DNAemia remained detectable but at a low level until lost of follow-up (48 days after 76 days of valganciclovir treatment discontinuation). Two SOT recipients (patients 4 and 7) developed 1-2 recurrent CMV disease episodes (follow-up: 9 months, range: 4-21 months) (Table 1). Median baseline viral load was 5.0 log copies/mL (range: 3.2-5.1 log copies/mL) in 5 patients without recurrent episode and 3.9 and 6.3 log copies/mL in patients with relapse. Median ganciclovir C_trough _during induction treatment was 1.5 mg/L (range: 0.8-3.3 mg/L) and C_3h _6.4 mg/L (range: 4.1-10.8 mg/L) in patients without relapse and 3.6 mg/L (range: 1.8-5.7 mg/L) and C_3h _6.5 mg/L (range: 5.3-6.7 mg/L) in patients with relapse, corresponding to ganciclovir exposure of 43.8 mg·h/L (32.7-74.3 mg·h/L) and 65.3 mg·h/L (44.3-117.9 mg·h/L), respectively. Overall, the median viral load decline of the 10 treatment courses in these 7 patients was -1.0 log/week with a wide variation ranging from 0 to -2.9 log/week.

Two patients had particularly complicate outcomes. Patient 4 was treated with oral valganciclovir a second time for a CMV syndrome 21 days after first therapy cessation and a third time for a CMV colitis 45 days after the second course discontinuation. CMV viremia decreased by -1.1 log copies/mL after 14 days, -3.0 log copies/mL after 15 days and -1.7 log copies/mL after 14 days during the first, second and third treatment, respectively (Figure 1). Trough ganciclovir concentrations and AUC determined during these two episodes remained at the therapeutic range despite dose reduction while the patient renal function deteriorated. This patient developed hepatitis with moderate hepatocellular insufficiency during the second episode of CMV disease, possibly related to high ganciclovir plasma levels, requiring treatment interruption. Anti-CMV IgG measured after the first and the second course of valganciclovir showed a seroconversion after the second CMV disease episode only (14 months after transplantation). CMV specific T-cell response was not assessed. He developed however a third episode of CMV disease. His condition deteriorated due to cardiac decompensation and respiratory failure. He died from a septic shock and multi-organ failure while the CMV viral load was decreasing.

Patient 7 had an unusual course upon treatment initiation, his viral load first increasing by 1.2 log copies/mL after 16 days of treatment and then decreasing by -2.4 log copies/mL over the second week of treatment even though ganciclovir plasma levels remained stable. Median ganciclovir plasma levels and systemic exposure (AUC) during induction treatment were 1.9 mg/L (range 1.8-2.6 mg/L) and 46.5 mg·h/L (range: 44.3-47.5 mg·h/L, 4 measures), respectively, thus showing very little changes. Dose of mycophenolate mofetil was reduced (1.5 g per day to 0.5 g per day) 5 days before valganciclovir treatment initiation as leucopenia was detected and stopped one week after valganciclovir treatment initiation. Dose of prednisone was increase from 5 mg per day to 10 mg and tacrolimus plasma level were maintained at 8 μg/L. This patient developed a second episode of CMV disease 62 days after first treatment discontinuation. CMV viral load stabilized at 4 log copies/mL before valganciclovir treatment start and became undetectable while on treatment (Figure 2). UL97 mutations were searched by gene sequencing but not detected. Anti-CMV IgM and IgG measured during the first and the second CMV episodes revealing low level of IgM from day 15 of first treatment course on while IgG remained undetectable until 4 months after the second treatment cessation (14.5 months after transplantation). CMV specific T-cell response assessed by ELISPOT assay after the first and second treatment (about 7 and 10.5 months after transplantation) showed low but rising numbers of IFN-γ-producing CMV-specific cells. Interestingly, this patient D+/R- for EBV developed also a high EBV viral load and did not build an EBV specific T cell response (Figure 2).

## Discussion

We report on 7 recipients D+/R- treated with valganciclovir for CMV late disease after prophylaxis (n = 6) and pre-emptively for CMV infection (n = 1) with monitoring of CMV viral load and ganciclovir plasma level. This small number of observations reflects the single centre nature of our study and the rather good efficacy of prophylaxis (which was prescribed to all patients except to few liver transplant recipients), decreasing the incidence of CMV disease among SOT recipients.

We observed a widely variable response with delayed CMV viremia load decrease in 1/7 recipient and recurrent infection in 2/7 patients. The rate of CMV viral load decrease after 1 week of treatment was in the range of those reported with iv ganciclovir [12] or oral valganciclovir [12,13], but viremia clearance was lower in our population including only D+/R- recipients. There was absolutely no indication that this variable response could be related to insufficient ganciclovir exposure, as shown by the result of ganciclovir plasma level monitoring revealing sufficient [14] or even rather higher concentrations in the patients with poor response. Ganciclovir needs to be bio-activated in infected cells into ganciclovir triphosphate to inhibit virus replication. In the absence of available clinical samples containing appropriate numbers of infected cells, ganciclovir plasma concentration is the only measurable surrogate of the actual active form concentration. Delayed viral clearance or recurrent infection seems thus more likely related to the absence of CMV cell-mediated immunity in a subset of patients experiencing primary infection while on immunosuppressive therapy [15]. However, the present study was not designed to assess the cell-mediated specific response to CMV. Emery et al, by comparing CMV replication dynamics in CMV-naïve and -experienced hosts, demonstrated that a higher drug efficacy was required to eliminate viral replication in non immune liver transplant recipients [16]. Additionally it has been shown that viral factors, such as infection with multiple CMV glycoprotein B genotypes, may also influence the response to antiviral therapy [17]. Interestingly, one patient experienced simultaneous primary infection by CMV and EBV, with delayed response to valganciclovir treatment and recurrent CMV infection. CMV primary infection has been reported to increase the risk of a EBV related PTLD in high risk (D+/R-) EBV recipients [18]. Conversely, one wonders whether EBV infection could influence the course of CMV infection.

## Conclusions

In conclusion, variable viral clearance could not be explained by a lower ganciclovir exposure in valganciclovir-treated patients, but was probably related to the immunological variability of seronegative recipients undergoing primary infection early after transplantation or after prophylaxis discontinuation or to viral factors.

## Competing interests

Roche Pharma Switzerland funded in part the present research program through a unrestricted research grant and gave lecture fees to PM.

## Authors' contributions

NP, MP and PM provided conceptual idea in drafting the paper. NP wrote the first draft of the paper and other coauthors contributed to the final draft. NP and PM were responsible for conducting the main study. OM and FL (in charge of these patients with JPV, MP and PM) informed them and asked for consent. NP and LAD validated an HPLC method to measure ganciclovir plasma level. NP interpreted ganciclovir plasma level. NP collected the data. NP and TB did the population pharmacokinetic analysis with NONMEM. Mutations in the CMV UL97 kinase gene were looked by RS. All authors participated in the data analysis and data interpretation. All authors read and approved the final manuscript.

## Pre-publication history

The pre-publication history for this paper can be accessed here:

http://www.biomedcentral.com/1471-2334/10/2/prepub

## Supplementary Material

Additional file 1 **Supplementary material**. Materials and methods for the determination of CMV antibody status, the assessment of CMV specific and EBV specific T-cell response and the detection of mutations in the CMV UL97 kinase gene are described in more detail. Click here for file
