# Supplementary material for: Variable viral clearance despite adequate ganciclovir plasma levels during valganciclovir treatment for cytomegalovirus disease in D+/R- transplant recipients
Source: BMC Infect Dis. 2010 Jan 6;10:2. doi: 10.1186/1471-2334-10-2 (PMC2820479; doi:10.1186/1471-2334-10-2)
Supplement: Additional file 1 — Supplementary material. Materials and methods for the determination of CMV antibody status, the assessment of CMV specific and EBV specific T-cell response and the detection of mutations in the CMV UL97 kinase gene are described in more detail. [file 1471-2334-10-2-S1.DOC]

**Supplementary material**

CMV antibody status of donor and recipient was determined for anti-CMV IgG by enzyme-linked fluorescent assay (ELFA, reference value: 4-6 EU/mL) (Vidas, BioMérieux, Marcy l’Etoile, France) and for EBV IgG by Viral Capsid Antigen immunofluorescence (VCA IF, reference value: titre 20) (Merifluor® EBV IgG IFA-IFT, Meridian Bioscience, Ohio, USA). Recipient CMV antibody were assessed for some patients during and after the treatment for IgM by enzyme immunoassay (EIA, reference value: index 0.9-1.1) (CMV-IgM-EIA test, Medac, Hambourg, Germany) and IgG by ELFA. EBV antibody were measured in one EBV D+/R- patient for IgM by VCA IF (reference value: titre 10) or by bead array immunoassay (VCA BAIA, reference value: 100-120 UA/mL) (AtheNA Multi-Lyte, Zeus Scientific, NJ, USA) and for IgG by VCA IF or by VCA BAIA (reference value: 100-120 UA/mL).

CMV specific and EBV specific T-cell response were assessed only in a single patient using interferon-γ (IFN-γ) enzyme linked immunospot (ELISPOT, limit of detection: 55 SFU/mio cells) (Becton and Dickinson company, NJ, USA).

Mutations in the CMV UL97 kinase gene were looked for in one patient. DNA was purified with the MagNApure LC instrument according to the manufacturer (Roche, magNApure DNA kit I) from either 200 μL whole blood or from an infected fibroblast cell culture inoculated with a gastric biopsy. Part of the UL97 region covering most of the known mutations associated with resistance to ganciclovir (codons 437-60912,13) was amplified by PCR using CMV_UL97M_F (TGCACGTTGGCCGACGCTAT: position 1308-1327 within the UL97 open reading frame) and CMV_UL97M_R (GCCGCCAGAATGAGCAGACA position 1837-1818 on the complementary strand of the UL97 open reading frame). PCR was done with 200 nmol/L each primer and 5 μL DNA in a 50 μL reaction containing 1.25 units Amplitaq Gold, 1.5 mmol/L MgCl2 in 1 x PCR buffer II (Applied Biosystems) supplemented with 4% Dimethyl sulfoxyde (Sigma), with the following cycling profile: 95°C for 9 min. followed by 40 cycles (95°/30'' 58°/1'30'' 72°/2') and a final extension step of 5' at 72°C.

Amplified DNA was analysed on a 2% agarose gel in the presence of ethidium bromide and the 530 base pairs amplicon purified with a PCR purification kit according to the manufacturer (Qiagen). DNA was then sequenced with the Big Dye terminator (v.1.1) chemistry according to the manufacturer (Applied Biosystems) using either PCR primer and subjected to capillary electrophoresis in an ABI3130XL instrument. Sequences were assembled from both strands and compared to the Genbank non redundant database using BLAST (Basic Local Alignment Search Tool, http://www.ncbi.nlm.nih.gov/blast/Blast.cgi).
